# Successful Closure of Outlet Muscular Ventricular Septal Defect Through Left Anterior Thoracotomy

**DOI:** 10.1016/j.atssr.2023.05.022

**Published:** 2023-06-15

**Authors:** Shinichiro Oda, Satoshi Fujita, Tomoki Ushijima, Tomoyuki Ono, Akira Shiose

**Affiliations:** 1 Department of Cardiovascular Surgery, Faculty of Medical Sciences, Kyushu University, Fukuoka, Japan

## Abstract

Closure of an adult doubly committed juxta-arterial ventricular septal defect (VSD) by left anterior mini-thoracotomy through the second or third intercostal space has been reported occasionally. However, the repair of an outlet muscular VSD, which is remote from the pulmonic valve annulus, by this approach remains controversial because of the limited operative field. Herein, we present the case of a 16-year-old boy weighing 65 kg with an outlet muscular VSD that was successfully repaired by a left anterior mini-thoracotomy approach. Our experience suggests that in select patients, outlet muscular VSD repair is technically feasible by left anterior thoracotomy.

Minimally invasive cardiac surgery (MICS) has gained popularity for the repair of mitral and aortic valves. The accumulation of experience with MICS techniques, such as peripheral cannulation and the use of specialized instruments, has enabled us to perform successful redo pulmonary valve replacement through left anterior mini-thoracotomy in 2 patients with repaired tetralogy of Fallot.^1^ This incision has also been found to be useful for the repair of adult doubly committed juxta-arterial ventricular septal defects (VSDs) through direct visualization and without the need for special surgical instruments.^2^^,^^3^ However, caution should be exercised in approaching outlet muscular VSDs, which are remote from the pulmonic valve annulus, because of the limited operative field in this incision.^4^ We present a technique for closure of outlet muscular VSDs through left anterior mini-thoracotomy.

A 16-year-old boy weighing 65 kg was diagnosed with outlet muscular VSD at 3 days of age. Despite the patient's being asymptomatic during follow-up, right coronary cusp prolapse and trivial aortic valve insufficiency developed at 16 years of age, and he was referred for surgery at our hospital. Preoperative computed tomography imaging demonstrated that the second intercostal space was nearest the ascending aorta, and the third intercostal space was nearest the VSD site (Figure 1). The diameter of the right internal jugular vein, right and left femoral arteries, and right and left femoral veins was 16.8 mm, 10.0 mm and 9.7 mm, and 9.9 mm and 9.5 mm, respectively. The patient was scheduled for VSD closure by a left anterior mini-thoracotomy approach.

**Figure 1:**
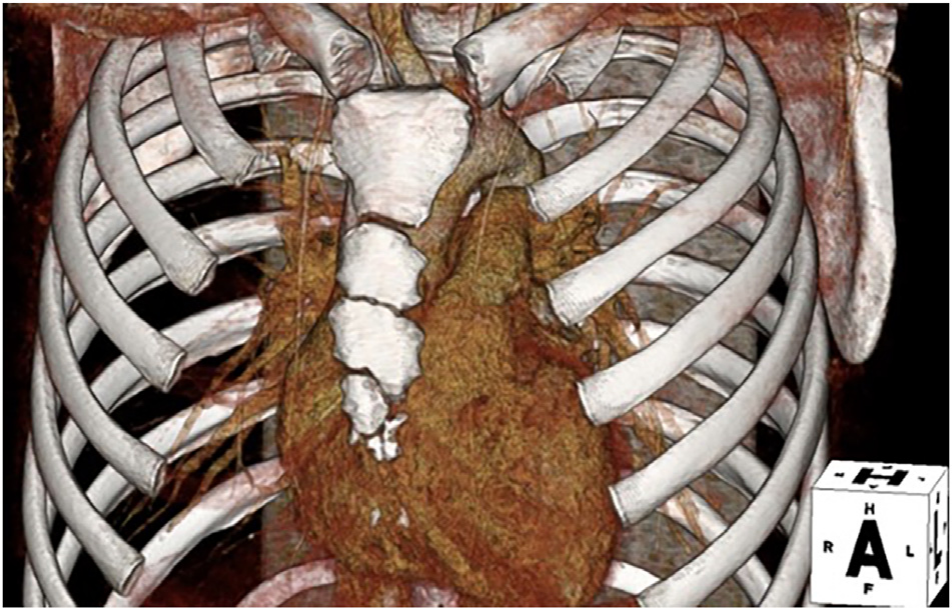
Preoperative 3-dimensional computed tomography image.

The surgical procedure was performed under general anesthesia using a double-lumen endotracheal tube. Intraoperative monitoring consisted of the left radial artery, right femoral artery, central venous pressure through the left jugular vein, cerebral and lower leg muscular near-infrared spectroscopy, nasopharyngeal and skin temperature, and urine output. Transesophageal echocardiography confirmed the absence of other intracardiac shunts. The patient was placed in the supine position with both arms pulled down and a pillow inserted under the scapula to extend the chest. The surgeon was positioned on the left side of the patient. A left anterior thoracotomy (5 cm in length) through the second intercostal space was performed (Figure 2). The second costal cartilage was divided to increase exposure. The pericardium was opened and suspended to expose the main pulmonary artery and the aortic root. A carbon dioxide insufflator tube was inserted through the same incision, and a wound retractor was used to provide maximum exposure (Figure 3).

**Figure 2:**
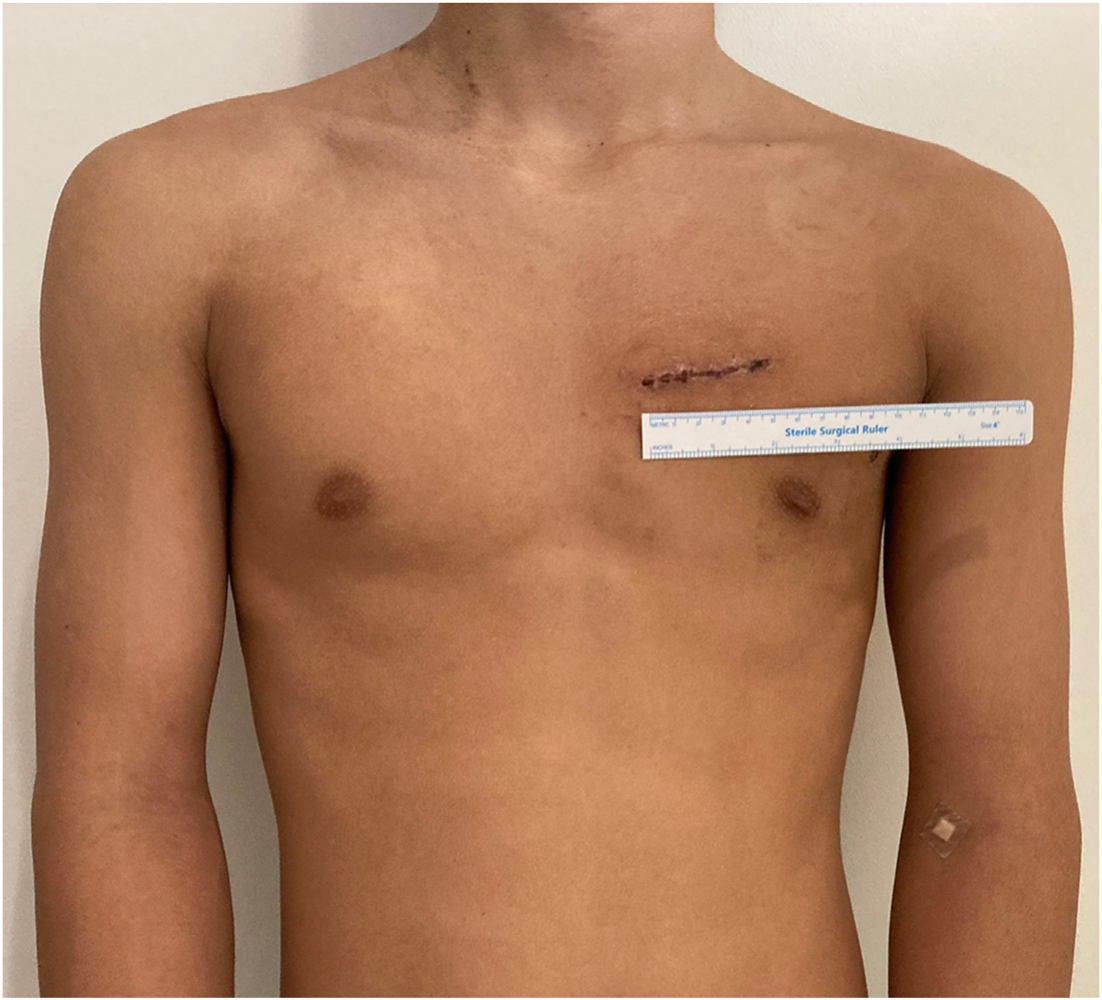
The left anterior wound.

**Figure 3:**
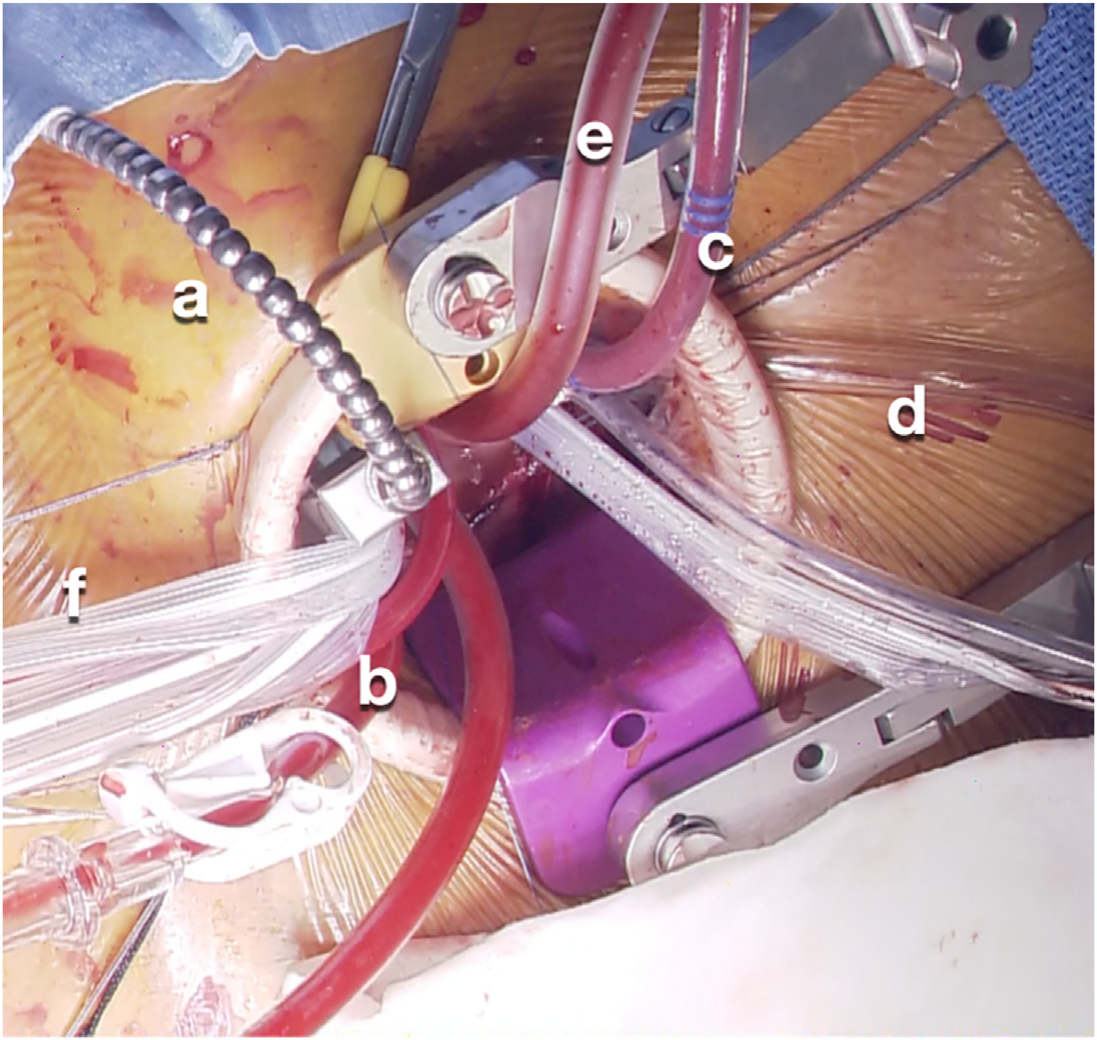
Operative field with an aortic cross-clamp (a), antegrade cardioplegia cannula (b), left atrial vent cannula (c), carbon dioxide insufflator tube (d), suction tube (e), and tape passed around the main pulmonary artery (f).

The left femoral artery was directly cannulated with a 17F Cortiva Bio-Medicus cannula (Medtronic), and a 23F long venous HLS cannula (Getinge) was inserted into the right femoral vein using the Seldinger technique. The tip of the venous cannula was advanced into the superior vena cava using fluoroscopy. A 16F left atrial vent cannula was inserted into the left atrial appendage. The left atrial vent cannula, the aortic cardioplegic cannula, and the Cygnet flexible aortic clamp (Vitalitec International, Inc) were inserted through the thoracotomy incision. After aortic cross-clamping and blood cardioplegic arrest, the main pulmonary artery was incised transversely and retracted downward. The outlet muscular VSD was visualized by bending the upper body and peering into the right ventricular outflow. With use of an ordinary needle holder, 6-0 double-armed sutures with buttressing spaghetti-type pledgets were placed on the superior margin of the defect. The traction on these stitches exposed the remaining posterior margin of the defect, through which the sutures were placed with forceps and a needle holder for MICS. The defect was closed with an expanded polytetrafluoroethylene patch (Gore-Tex; W. L. Gore & Associates). The stitches at the posterior margin of the defect were tied with a knot pusher. The main pulmonary arteriotomy was then closed. After deairing, the aortic clamp was released. The patient was smoothly weaned from the cardiopulmonary bypass and decannulated. The thoracotomy incision was then closed, and the patient was extubated within 105 minutes after the operation. Echocardiography revealed no residual VSD shunts or aortic valve insufficiency. The patient had an uneventful postoperative course.

## Comment

Intracardiac repair of the outlet muscular VSD through left anterior mini-thoracotomy with a transpulmonary approach was safely completed in this case. Compared with the doubly committed juxta-arterial VSD, the outlet muscular VSD lies far from the pulmonic valve annulus, raising concerns about inadequate exposure through a left anterior mini-thoracotomy approach.^4^ In our case, we had to bend our upper body to peek into the right ventricular outflow to visualize the defect, but our experience suggests that repair of outlet muscular VSDs through this approach is possible. We recommend using surgical instruments for MICS, which may not be required for closure of doubly committed juxta-arterial VSDs.^2^^,^^3^ The forceps and needle holder for MICS were kept at an appropriate angle from the incision. It is important to carefully plan the surgery and to conduct thorough preoperative examinations for the safety of the patient.

The following are the recommended steps for the left anterior thoracotomy approach:

• Computed tomography is routinely performed to accurately determine the locations of the main pulmonary artery, ascending aorta, intercostal space, and left atrial appendage. The diameters of the femoral artery and vein are also measured to confirm the size of the peripheral cannulations. If the groin vessels are inadequate for peripheral cannulation, left anterior mini-thoracotomy is contraindicated and median sternotomy is scheduled instead.
• To exclude other intracardiac shunts, transthoracic or transesophageal echocardiography is performed both preoperatively and perioperatively.
• Establishing safe cardiopulmonary bypass through peripheral cannulation is essential. To monitor for brain and lower leg muscle ischemia and venous stasis, we used a near-infrared wave monitoring system. To prevent arterial spasm, we applied a mixture of milrinone and lidocaine topically to the cannulated femoral artery. A left anterior mini-thoracotomy incision also allows direct ascending aortic cannulation, if necessary.
• To increase exposure, we divided the costal cartilage. The decision of which upper or lower costal cartilage should be divided is based on ease of access to the ascending aorta, which is a priority for safety.
• It is important to consider on preoperative computed tomography whether the insertion angle and length from the intercostal space to the VSD allow closure with MICS surgical instruments.

This approach offers several advantages compared with standard median sternotomy, including cosmetic effects, preservation of sternal integrity, and rapid postoperative recovery.^2^ In selected patients with outlet muscular VSD, left anterior mini-thoracotomy may provide therapeutic benefits.
